# Histone Deacetylase Inhibitors from Marine Invertebrates

**DOI:** 10.3390/biology9120429

**Published:** 2020-11-28

**Authors:** Claudio Luparello, Manuela Mauro, Vincenzo Arizza, Mirella Vazzana

**Affiliations:** Department of Biological, Chemical and Pharmaceutical Sciences and Technologies (STEBICEF), University of Palermo, 90128 Palermo, Italy; manuela.mauro01@unipa.it (M.M.); vincenzo.arizza@unipa.it (V.A.); mirella.vazzana@unipa.it (M.V.)

**Keywords:** histone deacetylase inhibitors, marine invertebrates, anticancer compounds, biomedical applications, Porifera, Cnidaria, Echinodermata

## Abstract

**Simple Summary:**

Histone deacetylases (HDACs) are enzymes that control gene expression and are involved in the onset of serious human pathologies, including cancer; hence, their inhibitors (HDACis) have received increased attention in recent years. It is known that marine invertebrates produce significant amounts of molecules showing active pharmacological properties and an extensive spectrum of biomedical applications. This review is focused on the description of the molecular, biochemical, and, where available, physiological aspects of marine invertebrate-derived compounds that possess HDACi properties, taking into consideration their possible utilization as treatment agents against different pathological states.

**Abstract:**

Histone deacetylases (HDACs) are key components of the epigenetic machinery controlling gene expression. They are involved in chromatin remodeling events via post-translational histone modifications but may also act on nonhistone proteins, influencing many fundamental cellular processes. Due to the key involvement of HDACs in serious human pathologies, including cancer, HDAC inhibitors (HDACis) have received increased attention in recent years. It is known that marine invertebrates produce significant amounts of secondary metabolites showing active pharmacological properties and an extensive spectrum of biomedical applications. The aim of this review is to gather selected studies that report the extraction and identification of marine invertebrate-derived compounds that possess HDACi properties, grouping the producing species according to their taxonomic hierarchy. The molecular, biochemical, and/or physiological aspects, where available, and modes of action of these naturally occurring HDACis will be recapitulated, taking into consideration their possible utilization for the future design of analogs with increased bioavailability and efficacy, less toxicity, and, also, higher isoform selectivity.

## 1. A Brief Insight into Histone Deacetylases and Histone Deacetylase Inhibitors

Histones are a group of proteins capable of interacting with DNA, thus forming the characteristic nucleosomal architecture of eukaryotic chromatin. Almost every biological process occurring in the nucleus, such as gene regulation, chromosome segregation, DNA replication, and repair, implies the subtle and precisely regulated adjustments of chromatin organization. Histone deacetylases (HDACs) are a key group of enzymes, highly conserved throughout evolution, involved in chromatin remodeling events via post-translational histone modification by catalyzing the removal of acetyl groups from lysine residues in their amino termini, thus determining chromatin condensation and transcriptional repression. These enzymes ay also act on non-histone proteins, thereby influencing many fundamental cellular processes, including microtubule dynamics and intracellular transport, metabolism, and aging [1]. Eighteen HDACs have been identified in humans and subdivided taxonomically into four classes, as extensively reviewed by [2,3]: 

Class I (HDAC1, -2, -3, and -8), whose members are expressed ubiquitously, localized into the nucleus, and involved in the control of cell viability and proliferation;

Class IIa (HDAC4, -5, -7, and -9) and IIb (HDAC6 and -10), whose members mostly show a tissue-specific expression profile and shuttle between the nucleus and the cytosol, with IIb enzymes controlling endothelial cell angiogenesis, cell proliferation, and migration, and T(reg) cell development and function;

Class III, including nuclear sirtuin (SIRT)1, -6, and -7, the mitochondrial SIRT3 and -4, and the cytosolic SIRT2 and -5, involved in diverse activities such as the deacetylation of transcription factors and β-catenin and the regulation of metabolism and the oxidative stress response; and

Class IV (HDAC11), often regarded as a hybrid of class I and II enzymes, shown to play a key role in the control of metabolism and obesity, the development of oligodendrocytes, and the functioning of the immune system.

The HDACs belonging to classes I, IIa, IIb, and IV are Zn^++^-dependent enzymes, whereas SIRTs require NAD^+^ for catalysis. 

The manifold regulatory roles played by HDACs account for their key importance in serious human pathologies, including cancer. Taking the aberrant gene expressions occurring in oncodevelopment into account, the involvement of HDACs is unsurprising and, also, on the basis of the known association of the enzymes with a number of well-characterized cellular oncogenes and oncosuppressor genes (e.g., Rb and p53), thereby altering the physiologic gene expression pattern and leading to cell hyperproliferation, invasion, and metastasis [4,5].

Histone deacetylase inhibitors (HDACis) belong to a large group of chemically different compounds, which includes complex molecules such as hydroxamates, cyclic tetrapeptides/epoxides, and benzamides, and relatively simple short-chain aliphatic acids, such as valproic acid and butyric acid [6,7,8,9,10,11]. This chemical heterogeneity implies a difference in the precise molecular mechanism of action that, for several members, is still poorly understood and, also, given that eighteen diverse HDACs exist in mammalian cells, with differing intracellular localizations, interactions with other proteins, and associations in multimolecular complexes.

HDACis are well-known antineoplastic compounds, the most potent to date being trichostatin A, active in vitro at nanomolar concentrations, which is a fermentation product of *Streptomyces* bacteria originally utilized as an antimycotic agent and later found to restrain the proliferation of cultured tumor cells [12]. The need for alternative HDACis, due to the difficulties and costs related to trichostatin A production, determined the introduction of a number of other inhibitors, such as butyrate, phenylbutyrate, depsipeptide, pyroxamide, suberoylanilide bishydroxyamide, valproic acid, and *N*-acetyldinaline in the clinical trials for anticancer activity, thus expanding the opportunities for epigenetic therapies [13]. In addition to the antineoplastic mechanism based upon reprogramming of the gene expression that ultimately suppresses cell proliferation and motility, HDACis have been proven to promote tumor cell death via apoptosis in a more powerful way when used in combination with other agents, e.g., [14], and, also, to inhibit endothelial cell growth and tumor angiogenesis [15,16]. Noteworthy, further applications of these compounds currently under study include the treatment of fungal infections and human neurological pathologies, such as Rett syndrome, Friedrich’s ataxia, Huntington’s chorea, and spinal muscular atrophy [17,18,19,20,21]. 

Seas and oceans, which cover three-quarters of the globe, host an enormous diversity of organisms, many of which are still unknown, and represent the underexploited richest source of bioactive marine natural products. Invertebrates, which account for more than 50% of the species colonizing the aquatic environment in Europe, are important environmental bioindicators [22,23,24,25]. They are known to produce significant amounts of secondary metabolites with unique chemical skeletons as molecular cues addressed to regulate very disparate biological activities, e.g., feeding; inter- and intraspecific signalizations; mating; predation; and defense from predators, competitors, pathogenic microorganisms, and UV radiation damage. These products contribute to the adaptation mechanisms to the specific life conditions in the greatly different marine ecosystems [26]. A large proportion of invertebrate-derived extracts and isolated compounds has shown active pharmacological properties, such as anticancer, antimicrobial, and anti-inflammatory, among others, with an extensive spectrum of biomedical applications that makes them already approved or prospective drugs of marine origin with promising results for different therapeutic purposes [27,28,29,30,31,32]. Within this scenario, the aim of this review is to gather selected studies that reported the extraction and identification of marine invertebrate-derived chemicals that possess HDACi properties and recapitulate the molecular, biochemical, and/or physiological aspects, where available, which are associated with the examined molecules. In particular, a focus will be put on the gene expression signatures associated with the exposure of different tumoral cytotypes to the distinct HDACis, when information is available. The matter of this review will be dealt with according to the taxonomic hierarchy of the producing invertebrate species, and the structural/zoological aspects of each type of organism will also be briefly discussed.

## 2. Porifera

Porifera is the oldest still-existing metazoan group, which comprises sponges, sessile aquatic organisms endowed with a very simple pattern of body organization constituted by a skin covering a collagenous matrix crossed by canals and microscopic chambers. A comprehensive picture of the global biodiversity of this phylum can now be obtained through the online World Porifera Database, which gathers all the taxonomic and distribution data of the species known so far [33]. 

### 2.1. Psammaplins

**Figure 1:**
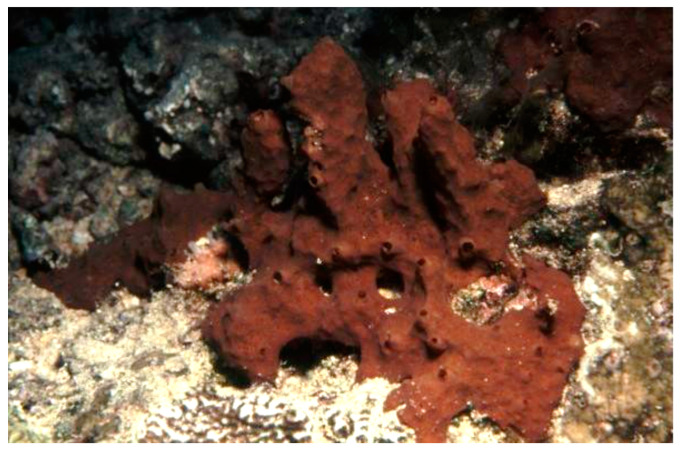
Specimens of *Aplysinella rhax* demosponge (CC BY 3.0 AU). https://bie.ala.org.au/species/urn:lsid:biodiversity.org.au:afd.taxon:e5607759-f60b-453a-b37a-30f3726d03c2#gallery.

*Aplysinella rhax* (Laubenfels, 1954; Demospongiae, Verongiida: Aplysinellidae), originally described as *Dysidea rhax* [34], is a demosponge commonly distributed in the Pacific Ocean that presents dendritic fibers with upright fleshy bulbs and an opaque, membranous, and optically smooth surface ornamentation overlying small clumps of fibers and forming an irregular and bumpy surface (Figure 1). 

**Figure 2:**
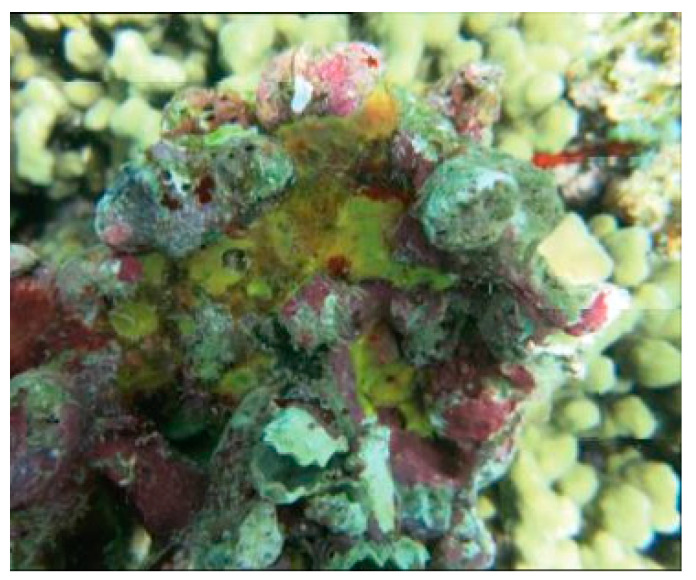
Specimen of *Pseudoceratina purpurea* demosponge [37].

*Psammaplysilla purpurea*, currently *Pseudoceratina purpurea* (Carter, 1880; Demospongiae, Verongiida: Pseudoceratinidae), is a subtropical, sessile, filter-feeder, and hermaphroditic demosponge populating the boulders and rock slabs in the lower eulittoral subzone of the Indian Ocean and the coasts of Madagascar, Saudi Arabia, and India. Its body consists of poorly developed spongin fibers with a collagenous choanosome, being irregularly spherical with an uneven surface that houses scattered, small, and sharp conules and prominent oscula (Figure 2) [35,36].

**Figure 3:**
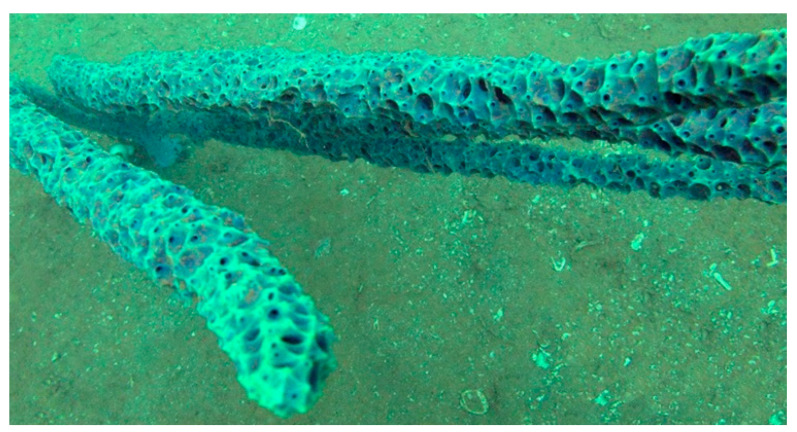
Specimens of *Dendrilla lacunosa* demosponge [38].

*Dendrilla lacunosa*, currently *Ernstilla lacunosa* (Hentschel, 1912; Demospongiae, Dendroceratida: Darwinellidae), was first described in Indonesia in 1912 and, 100 years later, found also in Western Australia and updated. This organism shows a typical aqua blue color and long structures reminescent of whips (Figure 3) [38].

**Figure 4:**
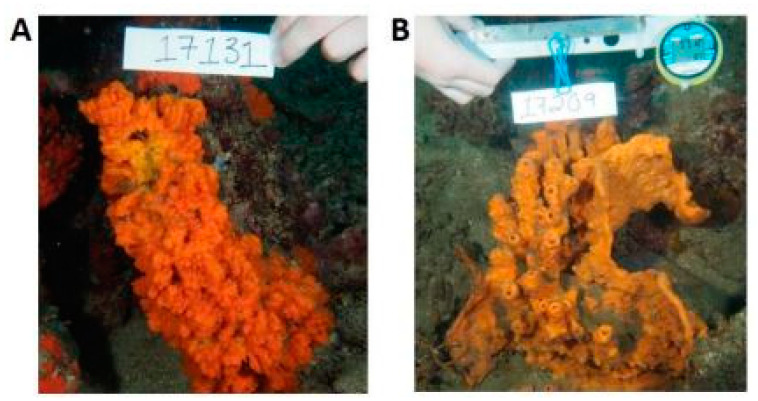
Two examples of species of the genus *Jaspis.* (**A**) *Jaspis coriacea* (Carter, 1886) and (**B**) *Jaspis splendens* (de Laubenfels, 1954) [41].

The species of the genus *Jaspis* (Demospongiae, Tetractinellida: Ancorinidae) comprise benthonic and suspension- or filter-feeder organisms typical of intertidal environments. Depending on the species, these sponges can have a wide distribution throughout Kenya, Sudan, Madagascar, South Korea, Australia, the Atlantic Ocean, and the Mediterranean Sea. In most cases, they display a massive spheroidal morphology, but, also, tubular or calyx structures are documented. These organisms are endowed with siliceous four-rayed spicules or, sometimes, with desmas without spicules and often show a very robust and strongly colored peripheral ectosomal layer (Figure 4) [39,40].

*Poecillastra wondoensis* (Sim and Kim, 1995; Demospongiae, Tetractinellida: Vulcanellidae) is a demosponge distributed in Korea, showing long oxeas with triaenes that include protriaenes longer than 2000 µm [42]. 

**Figure 5:**
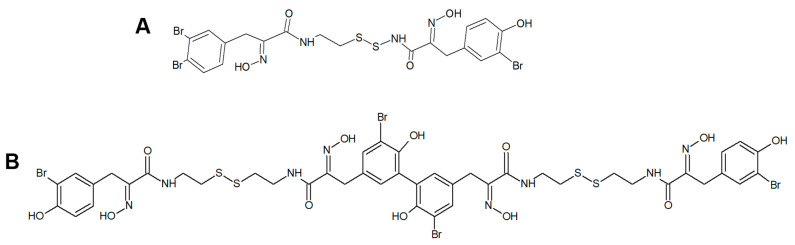
(**A**) Structures of psammaplin A (**A**) and bisaprasin (**B**).

The Porifera listed above have been proven to be good producers of psammaplins, a family of phenolic compounds whose HDAC inhibitory activity has been shown by experimental assays on different model systems in vitro. The first member isolated was the brominated tyrosine-derived psammaplin A (Figure 5A), initially described as an antimicrobial and antifungal agent acting through the impairment of DNA synthesis and chitinase enzymatic activity and then revealed as a HDACi acting via the coordination of a zinc ion in the catalytic pocket of HDAC, with a sulfhydryl group activated by a reducing agent [43,44,45,46]. Other members of the psammaplin group, i.e., psammaplin B–J and bisaprasin, were subsequently isolated and characterized, but only psammaplins A and F, the latter differing for a C_2_H_2_NO_3_ terminal group, a carboxylic acid moiety, a secondary amide functional group, and an *N*-substituted oxalamic acid group, and bisaprasin (Figure 5B) were proven to have HDACi properties when incubated with the ^3^H-acetylated human histone H4 peptide substrate [44,47]. In the following lines, the effects of psammaplins on neoplastic cell model systems are recapitulated.

Psammaplins and breast cancer cells: In light of molecular modeling data identifying several potential-binding sites for psammaplin A within the peroxisome proliferator-activated receptor γ (PPARγ) ligand-binding pocket, Mora et al. [48] demonstrated the psammaplin-induced activation of the receptor in a MCF-7 breast tumor cell-based reporter assay, followed by the promotion of apoptotic death at least in part mediated by the switch-on of the PPARγ-regulated gene expression. In 2012, Baud et al. [49] demonstrated the HDAC isoform selectivity of psammaplin A, which appeared to be 360-fold selective for HDAC1 over HDAC6 and more than 1000-fold less powerful towards HDAC7 and HDAC8, being the same selectivity profile maintained in MCF-7 cells as an in vitro model system. More recently, Zhou et al. [50] exposed both the estrogen-dependent T47D cells and different genetically characterized metastatic subclones of triple-negative MDA-MB231 cells specific to lung, bone, and brain to different psammaplins. They found a powerful inhibitory activity of the proliferation and three-dimensional invasive growth of tumor cells, except for the brain metastatic subclone, by the stereo isomers (*e*,*z*)-psammaplin A and (*e*,*e*)-psammaplin A compared to bisaprasin and psammaplins E and K. From a molecular point of view, psammaplin A was proven to trigger the activity of the hypoxia-inducible factor (HIF) and the upregulation of HIF target genes, such as cyclin-dependent kinase inhibitor 1A (*CDKN1A*) and vascular endothelial growth factor A (*VEGFA*; this only by the *e*,*e* isomer) and the downregulation of sirtuin-1 (*SIRT1)*, the latter leading to the increased p53 acetylation and autophagy-related gene expression and, ultimately, to autophagic cell death.

Psammaplin A and endometrial cancer cells: Ahn et al. [51] reported the inhibitory effect of the compound on Ishikawa endometrial cancer cells via cell cycle arrest at the G_1_ and G_2_/M phases and apoptosis promotion. Molecular events associated with the impairment of the cell cycle and the induction of apoptosis were the downregulation of cyclin D1, cyclin E, and CDK4 (involved in the block of G_1_ phase progression); cyclin A, cyclin B1, and CDK2 (involved in the block of G_2_/M phase progression); and the upregulation of p21^WAF1^, along with a decrease in the level of hyperphosphorylated pRb. 

**Figure 6:**
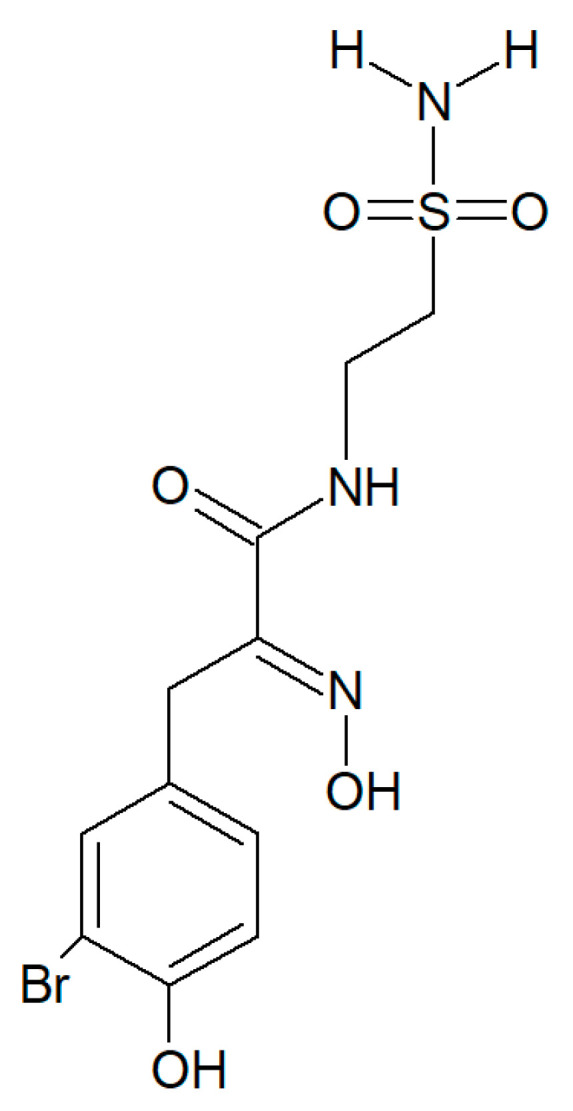
Structure of psammaplin C.

Psammaplin A and C and glioblastoma cells: psammaplin C (Figure 6) is a powerful inhibitor of carbonic anhydrase XII [52], whose activity is required to ensure an efficient efflux of chemotherapeutics by a P-glycoprotein pump in tumor cells. Salaroglio et al. [53] reported that carbonic anhydrase XII is overexpressed in glioblastoma stem cells and that a combination of psammaplin C and temozolomide, the latter being the first-line drug in glioblastoma treatment, rescues its efficacy against the highly chemorefractory stem component of the glioblastoma cell population. More recent data [54] demonstrated an even greater efficacy, in the order of subnanomolar carbonic anhydrase XII inhibitory activity, by a variant of the molecule endowed with a thiadiazole sulfonamide moiety replacing the ethyl sulfonamide one, which was able to inhibit also carbonic anhydrase IX, the other cancer-associated isozyme. 

Dealing with psammaplin A, a molecular study performed by Ratovitski [55] on U87-MG glioblastoma cells demonstrated the ability of the compound to induce the expression and phosphorylation of TP53 family members instrumental for the transcriptional activation of downstream target genes such as those involved in autophagy signaling, i.e., *ATG5* coding for the Autophagy-Related 5 protein and *UVRAG* coding for the UV Radiation Resistance-Associated protein. Confirmation of the autophagy flux-promoting activity was obtained by an electrophoretic analysis of the LC3B-I/LC3B-II shift. 

**Figure 7:**
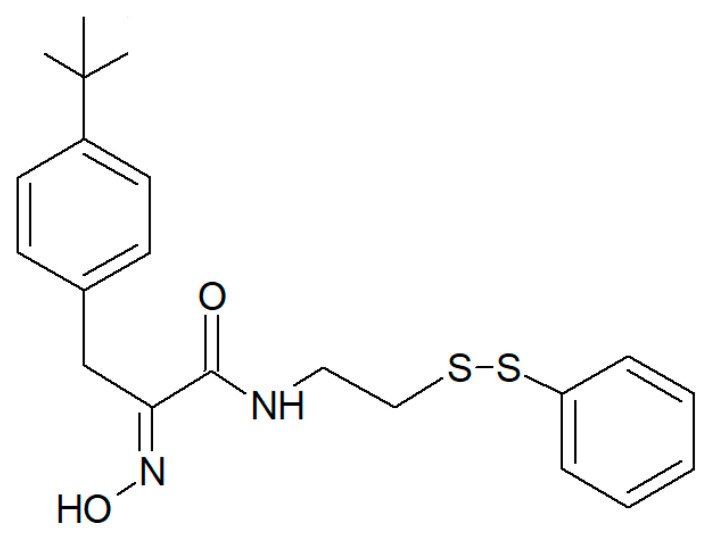
Structure of psammaplin A-3091 [56].

It must be mentioned that a number of experimental investigations, such as that of Mujumdar et al. [52] referenced above, have been focused on the synthesis and characterization of a series of psammaplin derivatives that might show a greater HDAC inhibitory efficacy and cytotoxic potential, although they provided mixed results. Among the most successful ones, the studies of Baud et al. [49] demonstrated the particularly potent activity of the (2*E*,2′*E*)-*N*,*N*′-(2,2′-Disulfanediylbis (ethane-2,1-diyl))bis(3-(3-bromo-4-methoxyphenyl)-2-(hydroxyimino)propanamide) variant towards MCF-7 breast cancer and A549 lung cancer cells. In addition, Byun et al. [56] reported the significant in vitro and in vivo anti-breast tumor and antimetastatic activity of psammaplin A-3091 (Figure 7), a heteromonomeric-structured analog with a tertiary butyl functional group able to specifically target *DOT1L*, coding for the disruptor of Telomeric silencing 1-like protein, involved in the regulation of both the epithelial-mesenchymal transition and the stem cell properties of breast cancer cells. 

### 2.2. Yakushinamides

**Figure 8:**
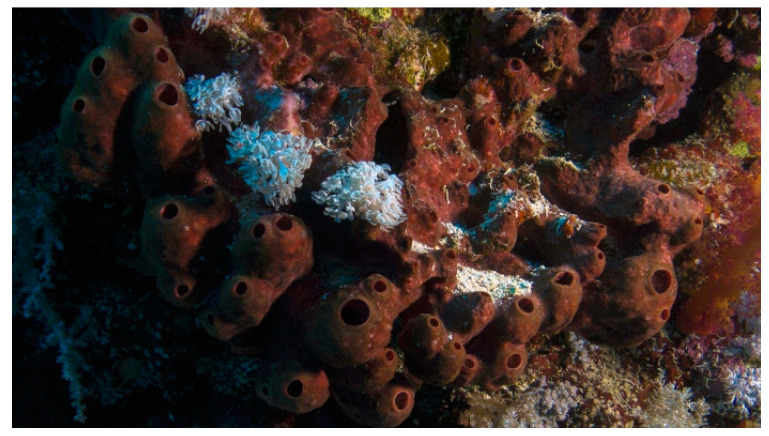
Image of a colony of *Theonella swinhoei* demosponges; http://www.marinespecies.org/aphia.php?p=image&tid=171264&pic=133237. Licensed under a Creative Commons Attribution- Share Alike 3.0 License.

*Theonella swinhoei* (Gray, 1868; Demospongiae, Tetractinellida: Theonellidae, Figure 8) is a sessile, hermaphrodite, and filter-feeder species typical of the Indo-West Pacific area, where it populates the tropical environments in a depth range between 20 and 188 m. Its covering dimensions range from 2.0 to 200 mm, and it is regarded as a remarkable component of the sponge population in coral reef communities, much studied because it represents a suitable microenvironment for the development of different types of autotrophic and heterotrophic organisms that inhabit different parts of its body [57].

**Figure 9:**
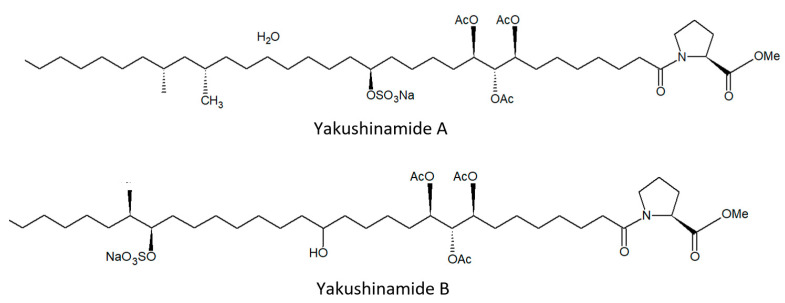
Structures of yakushinamide (**A**) and (**B**).

From this demosponge, Takada et al. [58] isolated two prolyl amides of polyoxygenated fatty acid, i.e., yakushinamide A (Figure 9), which displayed a moderate inhibitory effect on HDACs and sirtuins. In particular, yakushinamide A inhibited HDAC1, SIRT1, and -3 at 26, 16, and 79 μM, respectively, whereas yakushinamide B at 29, 75, 52, 34, 150, and 78 μM, respectively.

**Figure 10:**
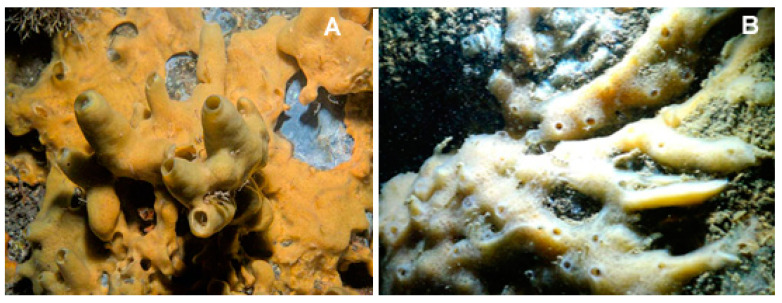
Two examples of species of the genus *Halichondria*. (**A**) *Halichondria panicea* (Pallas, 1766), taken from [60]. (**B**) *Halichondria bowerbanki* (Burton, 1930), taken from [61].

The species of the genus *Halichondria* (Fleming, 1828; Demospongiae, Suberitida: Halichondriidae, Figure 10) are massive and amorphous sponges typical of rocky substrates and with a cosmopolitan distribution. These organisms are filter-feeders, and some species of this genus can often develop on the carapace of crabs or on the shells of mollusks. Their internal and external skeletons consist of bundles of spicules arranged randomly. They can have a granular, smooth, or glassy shape, and their colors can vary according to the depth of the waters in which they are located, being white-grey in deep and greener in shallow waters thanks to the presence of light that allows the development of symbiotic algae in the latter case [59].

### 2.3. Halistanol Sulphates

**Figure 11:**
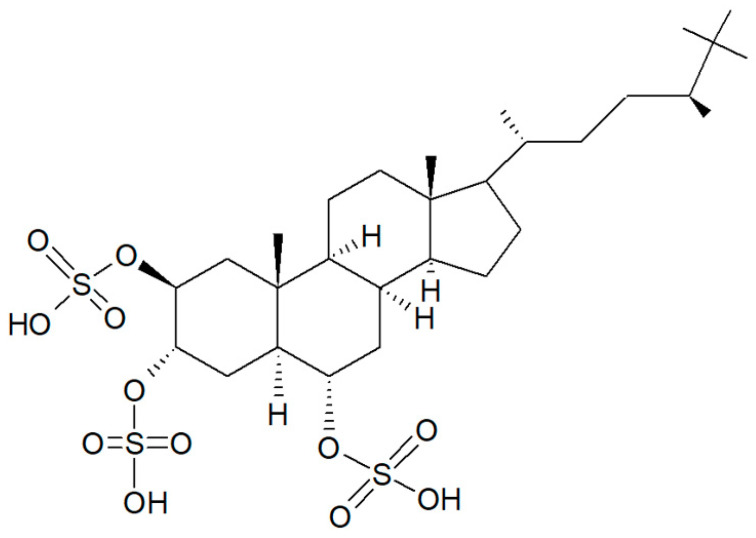
Structure of halistanol sulphate.

In the search for new SIRT inhibitors from marine sponges, from this species, Nakamura et al. [62] isolated the highly sulphated steroid halistanol sulphate (Figure 11) and two novel analogs, i.e., halistanol sulphate I and -J, differing from the parental molecule for methylene protons and the cyclopropyl ring in the side chain, respectively. When submitted to an in vitro SIRT1-3 inhibitory assay, these compounds were shown to be active, with half maximal inhibitory concentration (IC_50_) values of 45.9–67.9, 18.9–21.1, and 21.8–37.5 μM, respectively. On the other hand, they exerted no cytotoxic effects on HeLa cervical adenocarcinoma and P388 mouse leukemia cells at the concentration of 100 μM. Studies on the crystal structure of the SIRT3-halistanol sulphate complex indicated the existence of an allosteric site for enzyme inhibition far from the SIRT active site, substrate-binding site, and NAD^+^ cofactor-binding site, thus suggesting that steroid sulphates may be endowed with a good motif for the allosteric regulation of enzymes.

### 2.4. Azumamides

**Figure 12:**
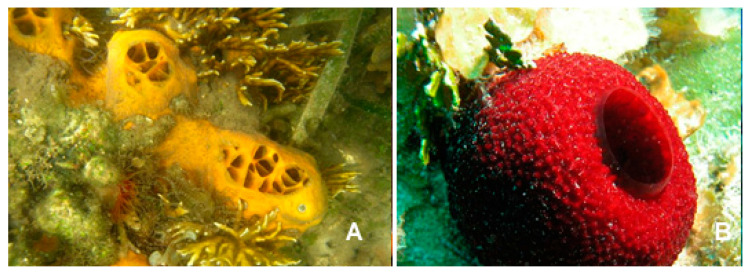
Two examples of species of the genus *Mycale*. (**A**) *Mycale laevis*, (Carter, 1882); CC0 1.0 (Public domain) https://serv.biokic.asu.edu/imglib/stri/misc/201605/13568_1464650224_web.jpg. (**B**) *Mycale laxissima* (Duchassaing and Michelotti, 1864); https://spongeguide.uncw.edu/imageinfo.php?img=319.

The species of the genus *Mycale* (Gray, 1867; Demospongiae, Poecilosclerida: Mycalidae; Figure 12) gather demosponges presenting smooth, subterminally constricted-style megascleres (“mycalostyles”) in combination with anisochelate microscleres [63].

**Figure 13:**
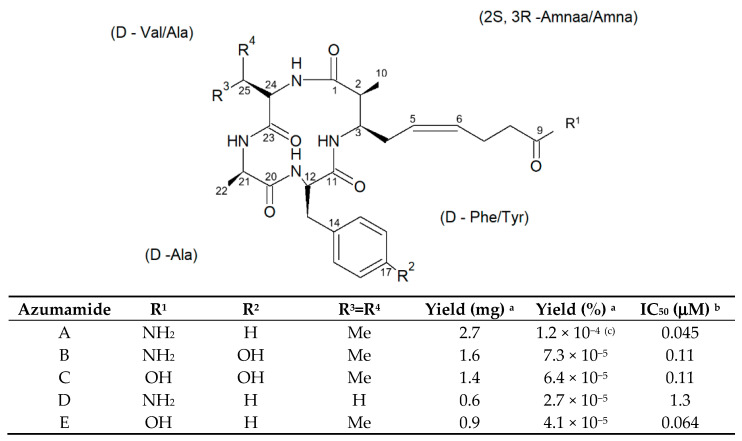
Structures of azumamides A-E. (a) Yield of azumamide isolated by extraction from a marine sponge. (b) Against the crude enzymes extracted from K562 cells. (c) Yield based on wet weight [65].

Among these, the species *Mycale izuensis* (Tanita and Hoshino, 1989) populates the Pacific Ocean, being especially typical of Japan, and is characterized by clusters of digitations up to 6-cm-high and 1.5 cm in diameter rising from a common base and bearing small apical oscules. Its body contains a skeleton of plumose spicule tracts that diverge and thin out towards the periphery [64]. From this marine organism, Nakao et al. [65] isolated five cyclic tetrapeptides named azumamides A-E endowed with HDACi activity in enzymatic assays (Figure 13). Azumamide A was initially also tested at the cell level, showing a moderate cytostatic effect on K562 cells and a significant antiangiogenic effect on mouse vascular progenitor cells. Subsequently, a total synthesis was reported for the sole azumamides A and E, and research interest was mainly focused on the greater HDACi efficacy of azumamide E, which appeared able to inhibit total HDACs from HeLa cell extracts with a much lesser IC_50_ (110 ± 33 nM) with respect to that of azumamide A (5800 ± 1200 nM) and showed selectivity for HDAC1-4. In particular, from a biological point of view, the compound proved to be a strong inhibitor of in vitro angiogenesis by mouse-induced pluripotent stem cells [66,67,68]. More recently, the total synthesis of all five natural azumamides, as well as their profiling towards the whole panel of HDACs, has been reported.

The data obtained revealed that azumamide C was a two-fold more potent inhibitor than azumamide E on the majority of HDAC isozymes. The observed discrepancy among the various evaluations of HDACi inhibitory activities was ascribed to the assay conditions largely affecting the results, underlining the need of their confirmation through parallel biological (e.g., antiproliferative or antiangiogenic) assays. On the other hand, surprisingly, given that the in vitro HDACi activity of azumamides B, C, and E were confirmed, no compound was able to reverse the chemoresistance caused by silencing of the proapoptotic *Bim* gene and, therefore, influence the growth of the Epstein−Barr virus (EBV)-infected human Burkitt’s lymphoma EB-3 cells differently from what was achieved upon treatment with HDACi SAHA [69,70]. This prompts a future investigation on azumamide analogs designed on the original scaffold but endowed with a more potent and selective ligand activity.

### 2.5. Cyclostellettamines and Dehydrocyclostellettamine

**Figure 14:**
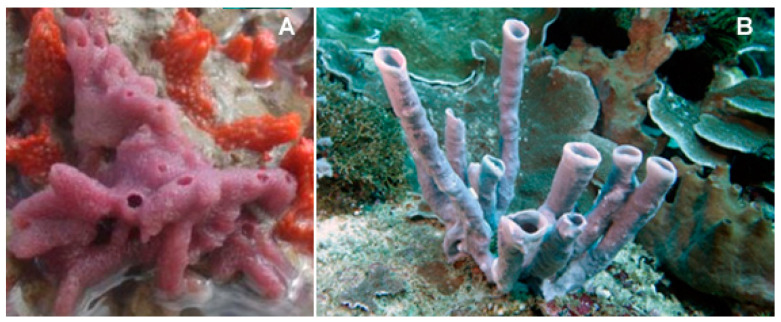
Two examples of species of the genus *Haliclona*. (**A**) *Haliclona implexiformis* (Hechtel, 1965); taken from [75]. (**B**) *Haliclona fascigera* (Hentschel, 1912); (CC BY-SA 3.0) http://www.marinespecies.org/photogallery.php?album=714&pic=132819.

The species of the genus *Haliclona* (Demospongiae, Haplosclerida: Chalinidae; Figure 14) are demosponges that live on poorly lit coral seaweed backdrops up to 40-m-deep in the Mediterranean Sea, the Western tropical Atlantic Ocean, the Caribbean Sea, the West Indies, and the Pacific coast. Some of these marine species are encrusting sponges with a fleshy but soft and fragile body. Their body is small and cone-shaped, with a maximum height of 8 cm, and their colonies can reach up to a diameter of 20 cm [71,72,73].

**Figure 15:**
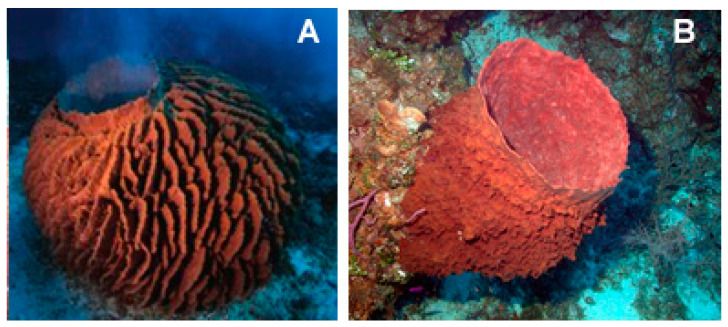
Two examples of species of the genus *Xestospongia*. (**A**) *Xestospongia testudinaria* (Lamark, 1815) taken from [76]. (**B**) *Xestospongia muta* (Schmidt, 1870) taken from https://commons.wikimedia.org/wiki/File:Reef3860_-_Flickr_-_NOAA_Photo_Library.jpg (CC BY 2.0).

The species of the genus *Xestospongia* (Demospongiae, Haplosclerida: Petrosiidae; Figure 15) are typical of the Indian and South Pacific Oceans and represent an outstanding source of secondary metabolites with different biological properties against human diseases. Within the *Xestospongia* genus, *Xestospongia vansoesti* (Bakus and Nishiyama, 2000) is a benthic species, filter, and suspension feeder commonly distributed at depths of 8–12 m but occurring also between 3 and 32 m in the Eastern Philippines and Indian and South Pacific Oceans. This sponge forms thick crusts but can also be a digitiform and is characterized by the production of copious brown mucus when handled or damaged. Its dense multispicular tracts contain oxeas, and the intact surface is smooth, but the dermal membrane is often missing, and, in this case, the individual is microrugose [74].

**Figure 16:**
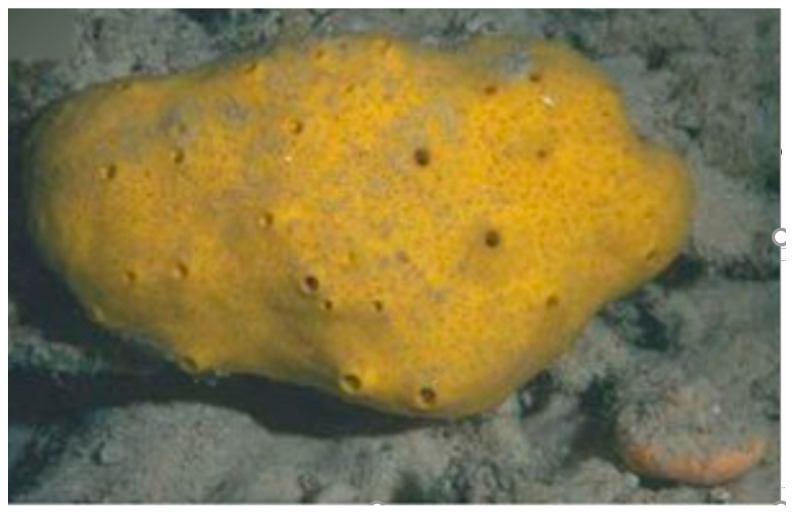
Specimen of *Petrosia alfiani* demosponge [75].

*Petrosia alfiani* (de Voogd and van Soest, 2002; Demospongiae, Haplosclerida: Petrosiidae; Figure 16) is distributed up to 40 m in the coral reef of Spermonde Archipelago, off the Southwest coast of Sulawesi in Indonesia, growing as thick and robust masses on coral blocks and rubble or coral sand. Its body is characterized by voluminous branches, such as massive globular or thick arms. Numerous small drains may be present on the sponge surface, which is smooth and bristly, with a consistency that can be stony hard or compressible [77]. 

**Figure 17:**
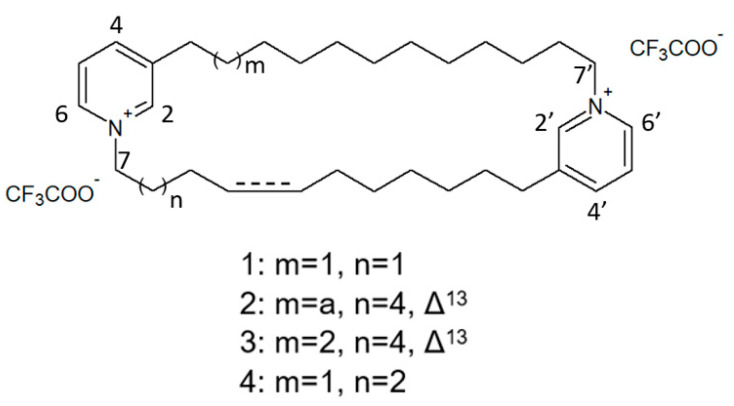
Structures of cyclostellettamine G (1), dehydrocyclostellettamine D (2), dehydrocyclostellettamine E, (3) and cyclostellettamine A (4) [78].

The species of the *Haliclona* and *Xestospongia* genus have proven to be good sources of natural HDACis. In 2004, Oku et al. [78] isolated cyclostellettamine A and three new cyclostellettamine alkaloids, i.e., cyclostellettamine G and dehydrocyclostellettamines D and E (Figure 17), from species of the genus *Xestospongia*, all displaying inhibitory activity with IC_50_ values in the range between 17 and 80 μM on HDAC preparations partially purified from K562 cells and exerting a moderate cytotoxic effect on HeLa human cervix carcinoma, P388 mouse leukemia, and 3Y1 rat fibroblastic cells. 

**Figure 18:**
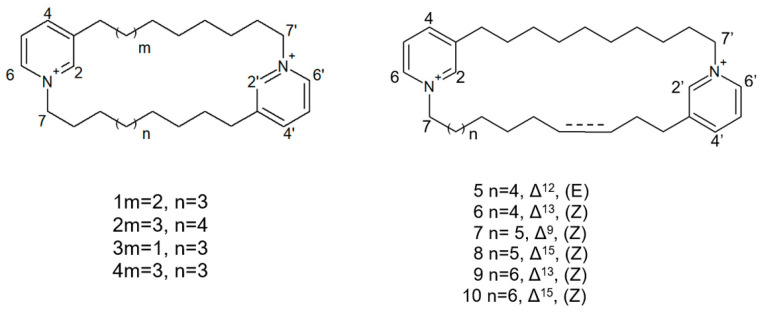
Structures of cyclostellettamine N (1), cyclostellettamine Q (2), and the other eight bis-1,3-dialkylpyridinium compounds extracted from species of the genus *Haliclona* (3–10).

More recently, Lee et al. [79] isolated eight novel cyclic bis-1,3-dialkylpyridinium compounds, as well as the two cyclostellettamines N and Q, from a species of the genus *Haliclona* (Figure 18), which exhibited a moderate doxorubicin-comparable cytotoxicity towards A549 lung cancer cells and, also, a diverse range of antimicrobial activity specifically directed against Gram-positive bacterial strains.

### 2.6. Halenaquinones

**Figure 19:**
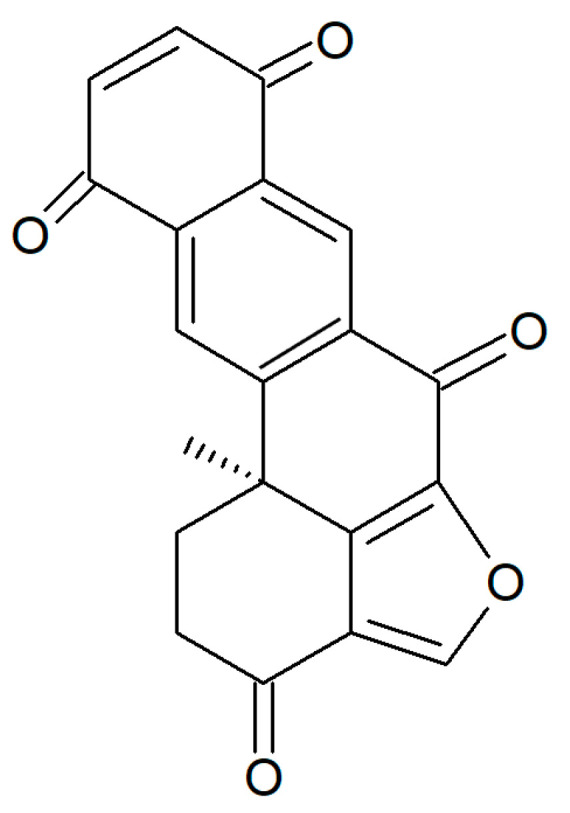
Structure of halenaquinone.

*X. vansoesti* and *P. alfiani* produce the polycyclic quinone-type metabolite halenaquinone (Figure 19) found to induce the inhibition of pan-HDACs and, in addition, also, topoisomerase IIα expression, the latter resulting in the switching-off of DNA replication. Dealing with the biological aspects, Shih et al. [80] demonstrated its cytotoxic activity on a number of cancer cell lines, being particularly remarkable against Molt 4 leukemia cells, in which viability suppression and apoptosis promotion following reactive oxygen species (ROS)-induced mitochondrial dysfunction was observed. The molecular signatures associated to the exposure of leukemia cells to the molecule were the upregulation of cytochrome c of the proapoptotic protein Bax, of the cytosolic hexokinase I, and of the active forms of caspase 8 and -9 and the downregulation of the antiapoptotic proteins Bcl-2, Bid, and cytosolic hexokinase II and of the phosphorylated (activated) forms of Akt, phosphatase, and tensin homolog (PTEN), glycogen synthase kinase 3 β (GSK3β), and phosphoinositide-dependent kinase-1 (PDK1). Of note, halenaquinone exhibited also a potent in vivo antileukemic effect in mice xenograft assays, reducing the weight and size of the tumor mass without affecting the total mice weight. 

In addition, Takaku et al. [81] demonstrated the inhibitory activity of the compound on DNA homologous pairing by direct binding to RAD51 enzyme, involved in the repair of DNA double-strand breaks, and subsequent likely competition with the double-strand DNA in the secondary DNA-binding site within the RAD51–single-strand DNA filament complex. More recently, Tsukamoto et al. [82] identified 65 as an inhibitor of the receptor activator of nuclear factor-κB ligand (RANKL)-induced upregulation of tartrate-resistant acid phosphatase (TRAP), which is involved in cell fusion, leading to the formation of multinucleated osteoclasts.

## 3. Cnidaria

Cnidarians constitute the first metazoan phylum endowed with a well-developed degree of tissue organization and gather polymorphic aquatic animals displaying radial or biradial symmetry and a single central body cavity (the “coelenteron”) with a mouth and tentacles but lacking an anus.

**Figure 20:**
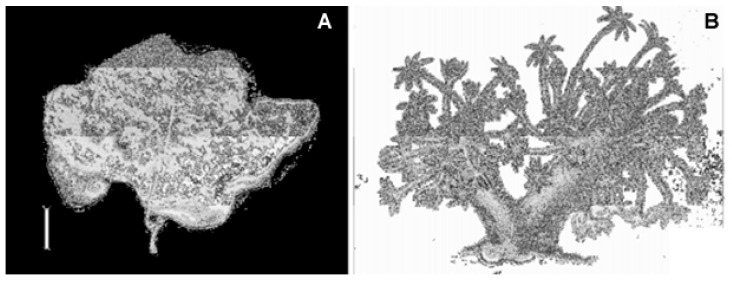
Specimen of *Xenia elongata* soft coral. (**A**) Photo of holotype in dry conditions. (**B**) Colony drawing from Dana (1846) [84].

*Xenia elongata* (Dana, 1846; Anthozoa, Alcyonacea: Xeniidae) is a photosynthetic soft marine coral that can be found in the Indian and Pacific Oceans and the Red Sea often clinging to the vertical surface of rocks between 0 and 10 m deep in order to obtain sufficient light and be exposed to strong currents. These organisms display a soft, fleshy, and feathered body with feather-shaped tentacles similar to fingers used to stir water with a constant and grabbing motion (Figure 20). For this reason, the species of this genus are commonly known as “pulse corals” [83].

**Figure 21:**
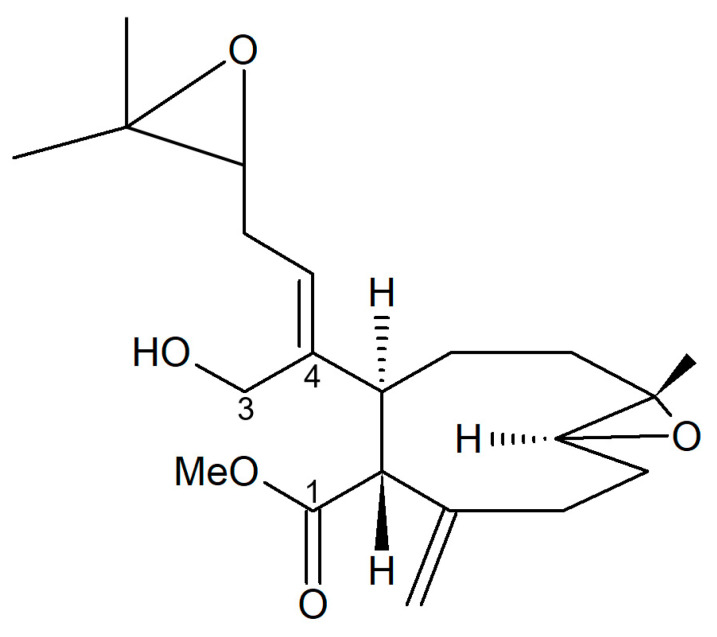
Structure of the diterpene extracted from *X. elongata* [85].

Andrianasolo et al. [85] isolated a new diterpene from *X. elongata* (Figure 21), which promoted the programmed death of immortalized apoptosis-competent W2 cells at a 1.2-μM concentration. Further enzymatic inhibition tests against the class I, -II A, and the -II B HDACs demonstrated that the compound specifically inhibited class II B HDAC6 with an IC_50_ of about 80 μM. In light of the data obtained, this molecule represents a new model structure of selective HDAC inhibitor that may be used for the development of novel HDAC isoform-targeting drugs.

## 4. Echinodermata

**Figure 22:**
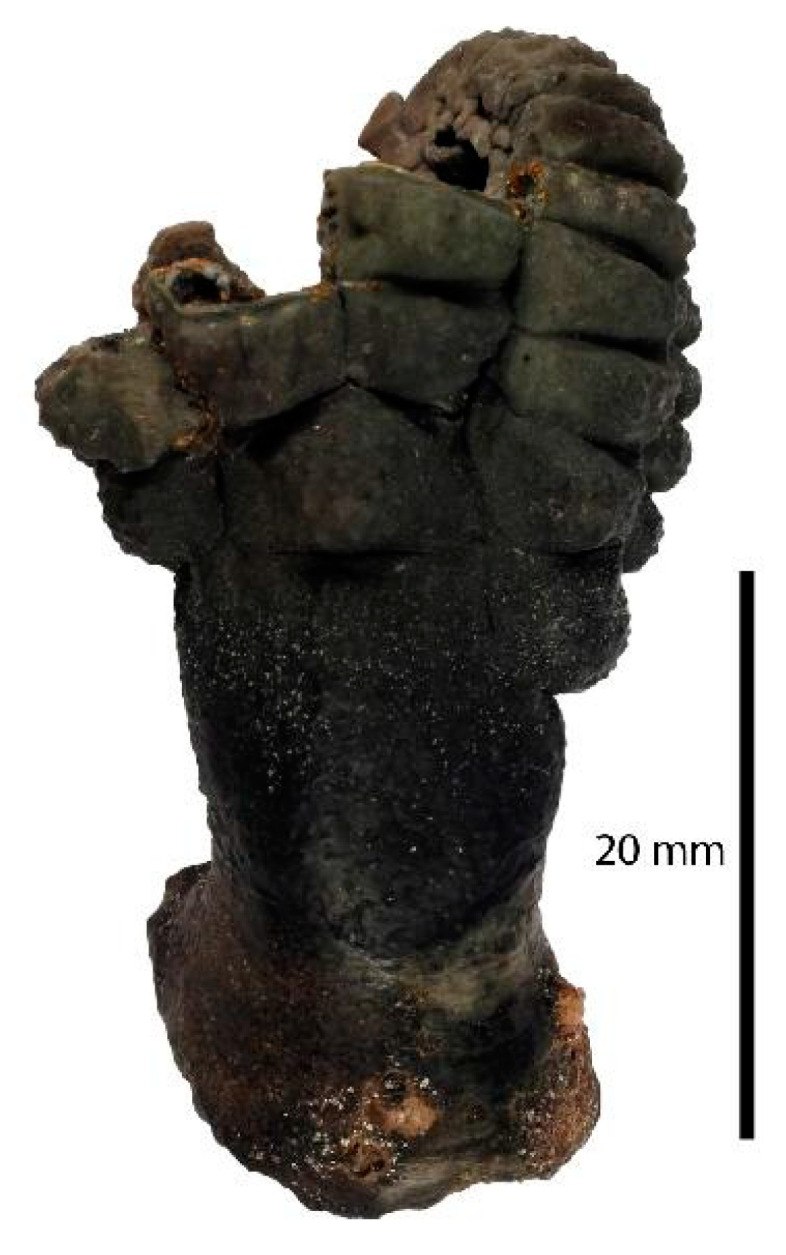
Specimen of *Holopus rangii* crinoid. Muséum national d’Histoire naturelle, Paris (France), Collection: Echinoderms (IE), Specimen MNHN-IE-2013-10116; http://coldb.mnhn.fr/catalognumber/mnhn/ie/2013-10116.

Echinoderms are marine invertebrates endowed with a hard, spiny covering or skin. *Holopus rangii* (Orbigny, 1837; Crinoidea, Cyrtocrinida: Holopodidae, Figure 22) is a large sessile but nonpedunculated crinoid populating the depths of Caribbean slopes and around Roatan Island, Honduras. It represents one of only eight extant species of Cyrtocrinida, an enigmatic invertebrate group diversified during the Jurassic age. Its skeleton is made up of thick plaques that are connected with sparse muscle joints. Its calyx is typically tubular, somewhat irregular, and the upper edge is grossly pentagonal. This organism is equipped with robust arms curved inwards with radials that can show different sizes [86].

**Figure 23:**
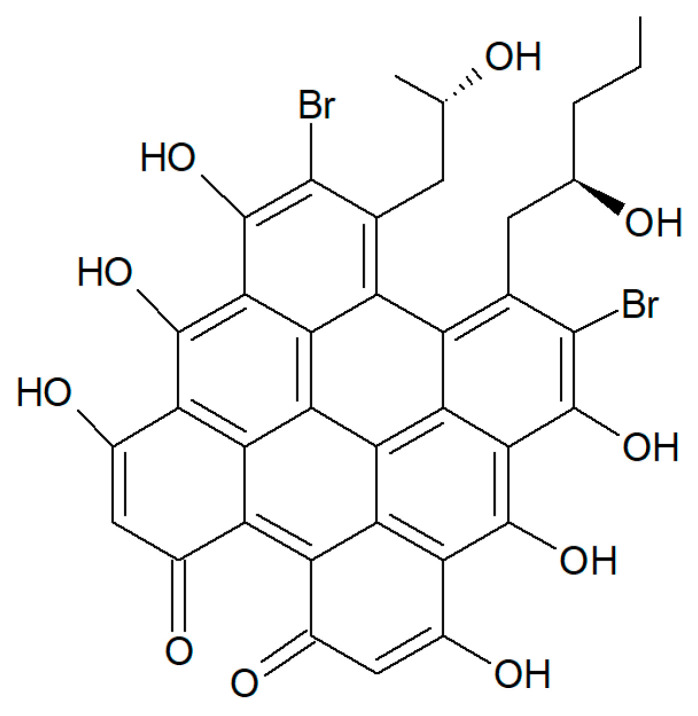
Structure of gymnochrome E.

From *H. rangii,* Kemami Wangun et al. [87] isolated a novel member of the phenanthrol perylenequinone family of natural products denominated gymnochrome E (Figure 23) that showed an inhibitory activity towards purified HDAC-1 with an IC_50_ of 10.9 μM and selectively restrained the growth of the multidrug-resistant NCI/ADRRes ovarian cancer cell line with an IC_50_ value of 3.5 μM while exerting no effect on PANC-1 pancreatic carcinoma and DLD-1 human colorectal adenocarcinoma cell lines. Moreover, the compound appeared endowed with an antimicrobic activity against *Staphylococcus aureus* and its methicillin-resistant strain with a minimum inhibitory concentration of 25 μg/mL but not against *Pseudomonas aeruginosa* and *Candida albicans*.

## 5. Conclusions

It is widely acknowledged that the enormous biodiversity of the marine habitats represents a source of immeasurable value for the isolation of very disparate bioactive secondary metabolites from bacteria, plants, and animals whose number is expected to increase rapidly, e.g., [88]. Among the enzymatic inhibitors searched in extracts from marine organisms, a focus has been put on HDACis for their wide range of biomedical applications. Natural inhibitors have been isolated from aquatic microorganisms [89,90], and, as documented by the studies presented in this review, also, marine invertebrates have contributed with a number of compounds displaying impairing properties of various potency towards HDACs, demosponges being the most investigated to date. Table 1 reports a schematic outline of the procedures used for the extraction, isolation, and identification of the HDACis from the marine organisms, whereas Table 2 summarizes, in a synoptic way, the invertebrate species, the isolated molecules, and their observed effects discussed in the text. It is worth mentioning that some marine species-derived molecules showing structural similarities with known HDACis failed to exert HDACi-referable cellular and molecular effects, as in the case of SAHA- and trichostatin A-resembling N-(4-guanidinobutyl)-2-(4-hydroxy phenyl)-2-oxo-acetamide isolated from the cnidarian hydroid Campanularia sp. [91]. Therefore, a thorough biological characterization of the novel compounds identified in the future, including the identification of the specific, if any, target cytotype(s), appears to be a necessary aspect for the subsequent development of efficacious prevention and/or treatment agents against different pathological states—among them, cancers. On the other hand, the unique and peculiar chemical scaffolds presented by the marine-derived HDACis may be successfully utilized for the design of analogs with increased bioavailability and efficacy, less toxicity, and, also, very interestingly, higher isoform selectivity.

## Figures and Tables

**Table 1:** Extraction, isolation, and identification procedures for the inhibitory histone deacetylases (HDACis) from marine invertebrates.

Compound	Extraction and Isolation	Identification	Ref.
Azumamides	Extraction in EtOH and MetOH, partitioning between water and Et_2_O, further extraction with *n*BuOH, octadecylsilane flash chromatography, gel filtration and octadecylsilane HPLC	Inhibition assay with crude enzymes extracted from K562 cells	[56]
Cyclostelletamine A and G Dehydrocyclostelletamine D and E from *Haliclona* sponges	Extraction with EtOH, partitioning between water and Et_2_O, further extraction with *n*BuOH, separation by octadecylsilane flash chromatography, further partitioning between CH_2_Cl_2_ and 60% MeOH, fractionation of the latter layer by octadecylsilane (aqueous MeOH), TSK G3000S (aqueous MeCN–5% AcOH), and Sephadex LH-20 (CHCl_3_–MeOH, 1:1) chromatography, further purification by HPLC on phenylethyl-SiO_2_ (MeCN–H_2_O, 52:48, containing 0.1% TFA) and finally by HPLC on C30-SiO_2_ (aqueous n-PrOH containing 0.1% TFA)	Inhibition assay with HDACs partially purified from K562 cells and [^3^H] acetyl histoneH4 peptide	[64]
Diterpene from *X. elongata*	Extraction with CH_2_Cl_2_ and then with MeOH, fractionation of the polar crude organic fraction by solid phase extraction cartridge (Reverse-phase C18), chromatography of the fraction eluting with 15% H_2_O and 85% MeOH on analytical RP HPLC (Phenomenex luna C8) using a gradient elution (starting with 80% water and 20% CH_3_CN	Inhibition assay with isolated HDACs and fluorogenic substrates	[69]
Gymnochromes E and F	Successive extractions with EtOH and EtOAc/EtOH (1:1), fractionation on a C-18 stationary phase using vacuum column chromatography, further purification using reversed phase HPLC	Inhibition assay with HDACs partially purified from H1299 cells and [^3^H] acetyl histoneH4 peptide	[70]
Halenaquinone	Extraction with MeOH, partitioning between CHCl_3_ and water, dissolution of the evaporated organic layer into water⁄ MeOHl (1:9), extraction with CHCl_3_, fractionation and purification by reversed-phase HPLC on a C18 column with aqueous acetonitrile (water:acetonitrile 7:3–3:7, linear gradient	Commercial HDAC Inhibitor Drug Screening assay (BioVision, Milpitas, CA, USA)	[66,67]
Halistanol sulphates	Extraction with MeOH, CHCl_3_, and then, *n*-BuOH, Kupchan procedure to yield an aqueous MeOH layer, separation by octadecylsilane flash chromatography (water/MeOH = 100/0, 80/20, 50/50, 30/70, 0/100, and CHCl_3_/MeOH/water = 6/4/1), separation of the fraction eluting with water/MeOH = 50/50 by reverse-phase HPLC	SIRT1–3 inhibitory tests by electrophoretic mobility shift assay	[54]
Psammaplins and bisaprasin	Extraction with 50% MeOH in CH_2_Cl_2_, separation by Sephadex LH-20 column (eluted with CH_2_Cl_2_/MeOH, 50:50), separation of the active fraction by a semi-preparative HPLC (C18(2), isocratic 63% MeOH in water)	Commercial luminescent assay (HDAC-Glo™, Promega, Madison, WI, USA)	[45]
Yakushinamides	Extraction with MeOH and EtOH, partitioning with CHCl_3_ and water and, then, between 90% MeOH and *n*-hexane, separation of the CHCl_3_ layer by octadecylsilane flash chromatography using a stepwise gradient elution (20−70% MeOH, 70−90% MeCN, and MeOH), separation of the active fraction by chromatography on silica gel using a stepwise gradient elution (CHCl_3_−MeOH−water, 98:2:0, 9:1:0, 8:2:0.1, 7:3:0.5, 6:4:1, and 5:5:1), separation by RP-HPLC using two columns connected in tandem with eluent consisting of 8:2 MeOH−phosphate buffer, purification by RP-HPLC with 5:5 MeCN−phosphate buffer	Inhibition assay with recombinant SIRTs and fluorogenic substrates	[52]

**Table 2:** Properties of HDACis from marine invertebrates.

Phylum	Species	Compounds	Effects	Ref.
**Porifera**	*Aplysinella rhax* *Pseudoceratina purpurea sub Psammaplysilla purpurea* *Ernstilla lacunose sub Dendrilla lacunosa* *Jaspis* sp. *Poecillastra wondoensis*	psammaplin A (*e,z*)-psammaplin A (*e,e*)-psammaplin A psammaplin E psammaplin K	Activation of peroxisome proliferator-activated receptor γ (PPARγ) in MCF-7 cells and promotion of apoptotic death HDAC isoform selectivity Powerful inhibition of the proliferation and three-dimensional invasive growth of tumor cells, stimulation of the activity of hypoxia-inducible factor (HIF) and upregulation of HIF target genes Growth inhibition and apoptosis promotion on Ishikawa endometrial cancer cells	[48] [49] [50] [51]
		psammaplin A psammaplin C psammaplin A-3091	Powerful inhibition of carbonic anhydrase XII Anticancer effect against the highly chemorefractory stem component of glioblastoma cells Stimulation of the expression and phosphorylation of TP53 family members Cytotoxicity towards MCF-7 breast cancer and A549 lung cancer cells Significant in vitro and in vivo anti-breast tumorigenesis and antimetastatic activity	[52] [53] [55] [49] [56]
	*Theonella swinhoei*	yakushinamide A	Moderate inhibitory effect on HDACs and sirtuins	[58]
	*Halichondria* sp.	halistanol sulphate halistanol sulphate I halistanol sulphate J	Inhibitory activity on SIRT1-3	[62]
	*Mycale izuensis*	azumamides A-E azumamide A azumamide E azumamide C	Moderate cytostatic effect on K562 human leukemia cells and significant anti-angiogenic effect on mouse vascular progenitor cells. Inhibitory activity on total HDACs from HeLa cell extracts with selectivity for HDAC1-4. Strong inhibition of in vitro angiogenesis by mouse induced pluripotent stem cells	[65] [66,67,68]
	*Haliclona* sp.	cyclostellettamine N cyclostellettamine Q	Moderate doxorubicin-comparable cytotoxicity towards A549 lung cancer cells and antimicrobial activity against Gram-positive bacteria	[79]
	*Xestospongia* sp.	cyclostellettamine A cyclostellettamine G dehydrocyclostellettamine D dehydrocyclostellettamine E	Moderate cytotoxic effect on HeLa human cervix carcinoma, P388 mouse leukemia, and 3Y1 rat fibroblastic cells	[78]
	*Xestospongia vansoesti*	halenaquinone	Inhibitory activity on DNA repair and on receptor activator of nuclear factor-κB ligand-mediated osteoclast maturation	[58,81]
	*Petrosia alfiani*	polycyclic quinone-type metabolite halenaquinone	Inhibition of pan-HDACs and topoisomerase IIα expression, cytotoxic activity against Molt 4 leukemia cells and in vivo antileukemic effect in mice xenograft assays	[80]
**Cnidaria**	*Xenia elongata*	diterpene	Induction of programmed death of immortalized apoptosis-competent W2 cells and inhibition of class II B HDAC6	[85]
**Echinodermata**	*Holopus rangii*	gymnochrome E	Inhibitory activity towards purified HDAC-1 and selective growth impairment of the multidrug-resistant NCI/ADRRes ovarian cancer cell line. Antimicrobic activity against *Staphylococcus aureus* and its methicillin-resistant strain	[87]
